# Data on synthesis, ADME and pharmacological properties and early safety pharmacology evaluation of a series of novel NURR1/NOT agonist potentially useful for the treatment of Parkinson's disease

**DOI:** 10.1016/j.dib.2019.104057

**Published:** 2019-05-27

**Authors:** André Malanda, Pierre-Yves Abécassis, Pascal Barnéoud, Pascale Brunel, Véronique Taupin, Xavier Vigé, Dominique Lesuisse

**Affiliations:** a Rare and Neurologic Disease Research, Sanofi, 1 av Pierre Brossolette, F-91935 Chilly Mazarin, France; b IDD, Sanofi, 1 av Pierre Brossolette, F-91935 Chilly Mazarin, France; c DSAR, Sanofi, 13 quai Jules Guesde, F-94403 Vitry-sur-Seine, France; d Preclinical safety, Sanofi, 3, digue d’ Alfortville, F- 94140 Alfortville, France; e Translational Science, Sanofi, 1 av Pierre Brossolette, F-91935, Chilly Mazarin, France

## Abstract

This article describes the chemical synthesis, ADME and pharmacological properties and early safety pharmacology evaluation of a series of novel Nurr1/NOT agonist. It is meant as a support to an article recently published in Bioorganic and Medicinal chemistry Letters and entitled “Development of a novel NURR1/NOT agonist from hit to lead and candidate for the potential treatment of Parkinson's disease” [1] and presenting the discovery, scope and potential of these new ligands of these nuclear receptors.

**Table:** Specifications Table

Subject area	*Medicinal chemistry, biology, pharmacology*
More specific subject area	*Agonists of the NURR1/NOT nuclear receptor, potentially useful in the treatment of Parkinson's disease.*
Type of data	*Experimental procedures for the synthesis of the compounds and their evaluation in all early ADME assays along with biochemical assays, pharmacological and early safety pharmacology evaluation.*
How data was acquired	*For structural confirmation NMR spectra were measured for all compounds. The NMR spectra were recorded on a Bruker Avance II* 400 MHz *spectrometer using* 5 mm *PAPPO-BB probe head at 303K temperature in DMSO-d6 solution. The chemical shifts were referred to tetramethylsilane (δ, ppm) and the coupling constants in Hertz (s = singlet, d = doublet,t = triplet, q = quadruplet, m = multiplet).* *LCMS analyses were performed on a Waters Acquity-SQD UPLC-MS system in positive and negative ESI mode using a short gradient method on an Acquity UPLC Cortecs C18+, 1.*6 μm*, 2.1 × 50mm chromatographic column (40°C, flow rate: 1.*0 mL/min*, 2–98% ACN in* 3 min*, 0.1% formic acid in both eluents).* *The melting points were measured with a Büchi Melting Point B-545 (Mp = Melting point).*
Data format	*Analyzed*
Experimental factors	*For structural confirmation NMR spectra were measured for all compounds. The NMR spectra were recorded on a Bruker Avance II* 400 MHz *spectrometer using* 5 mm *PAPPO-BB probe head at 303K temperature in DMSO-d6 solution. The chemical shifts were referred to tetramethylsilane (δ, ppm).* *LCMS analyses were performed on a Waters Acquity-SQD UPLC-MS system in positive and negative ESI mode using a short gradient method on an Acquity UPLC Cortecs C18+, 1.*6 μm*, 2.1 × 50mm chromatographic column (40°C, flow rate: 1.*0 mL/min*, 2–98% ACN in* 3 min*, 0.1% formic acid in both eluents).* *The melting points were measured with a Büchi Melting Point B-545.*
Experimental features	*Chemical syntheses and characterization of all compounds, full ADME profile of the final compounds,* in vitro *and in vivo characterization of the best agonists, early cardiac safety data.*
Data source location	*Sanofi, 1 av Pierre Brossolette Chilly Mazarin, F-91935*
Data accessibility	*Data is with this article; For the sake of clarity, compounds numbering will be kept the same as in the related research article referenced below.*
Related research article	*Development of a novel NURR1/NOT agonist from hit to lead and candidate for the potential treatment of Parkinson's disease, Bioorganic and Medicinal Chemistry “in press.”**[1]*

**Table:** 

**Value of the data** This report features experimental conditions and protocols used for the synthesis and evaluation of novel agonists of the NURR1/NOT nuclear receptor. The reasons why this data is of value for the community are the following: • These compounds are a potentially exciting and high impact development, especially for the neurodegeneration field, because of the importance of the drug target and the potential novelty and high impact of the proposed therapeutic strategy. • While selective activation of RXR/Nurr1 heterodimers through the targeting of the RXR LBD as a potential monotherapeutic strategy has recently been established [2], the selective activation of RXR/Nurr1 using small molecules agonists targeting Nurr1 offers the potential for improved selectivity and therefore improved therapeutic index. • The full scope of these compounds has not been broadly investigated and access to their preparation will enable the scientific community to perform further explorations in various indications pertaining to Parkinson's disease, Alzheimer's disease or other neurodegenerative diseases.

## 1 Data

**Scheme 1:**
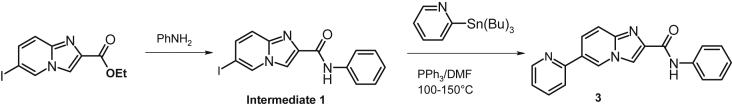
Synthesis of N-Phenyl-6-(pyrid-2-yl)imidazo[1,2-a]pyridine-2-carboxamide (cpd 3) [4].

**Scheme 2:**
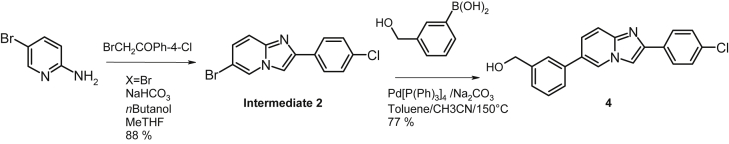
Synthesis of (3-[2-(4-Chlorophenyl)imidazo [1, 2-a]pyridin-6-yl]phenyl)methanol (cpd 4) [6].

**Scheme 3:**
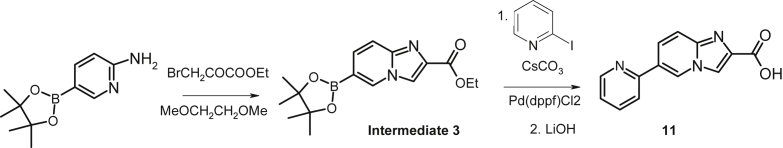
Synthesis of 6-(pyrid-2-yl)imidazo[1,2-a]pyridine-2-carboxylic acid (cpd 11) [7].

**Scheme 4:**
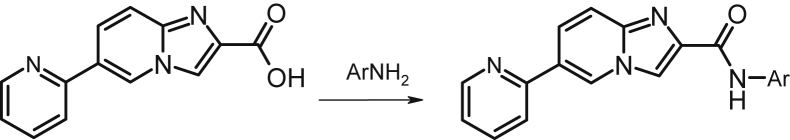

**Scheme 5:**
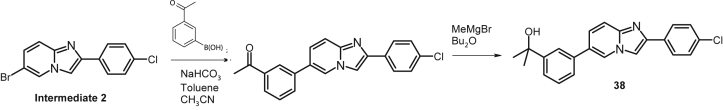
Synthesis of 2-(3-[2-(4-Chlorophenyl)imidazo[1,2-a]pyridin-6-yl]phenyl)propan-2-ol (cpd 38) [6].

**Scheme 6:**
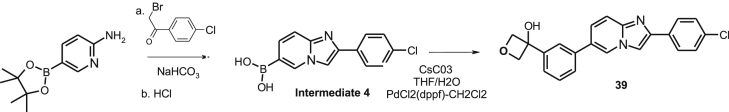
Synthesis of 3-(3-(2-(4-chlorophenyl)imidazo[1,2-a]pyridin-6-yl)phenyl)oxetan-3-ol (cpd 39).

**Scheme 7:**
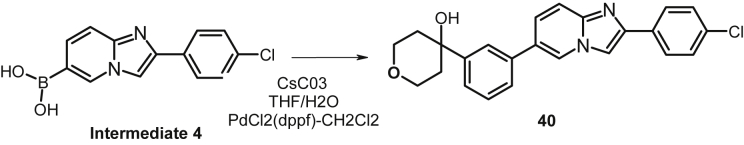
Synthesis of 4-(3-(2-(4-chlorophenyl)imidazo[1,2-a]pyridin-6-yl)phenyl)tetrahydro-2H-pyran-4-ol (cpd 40).

**Scheme 8:**
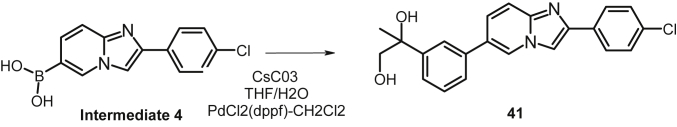
Synthesis of 2-(3-(2-(4-chlorophenyl)imidazo[1,2-a]pyridin-6-yl)phenyl)propane-1,2-diol (cpd 41).

**Scheme 9:**
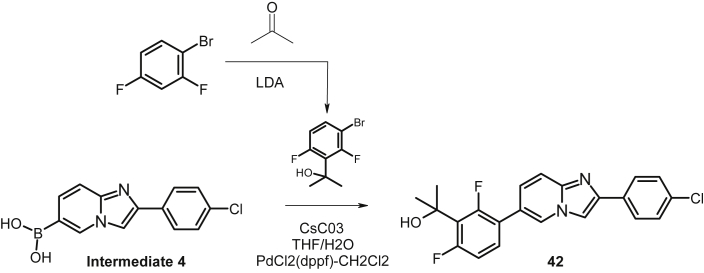
Synthesis of 2-{ 3-[2-( 4-Chlorophenyl)imidazo[1,2-a]pyridin-6-yl]-2,6-difluoro-phenyl}propan-2-ol (cpd 42) [9].

**Scheme 10:**
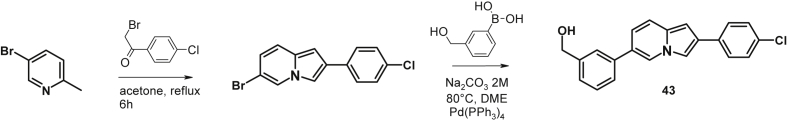
Synthesis of (3-(2-(4-chlorophenyl)indolizin-6-yl)phenyl)methanol (cpd 43).

**Scheme 11:**
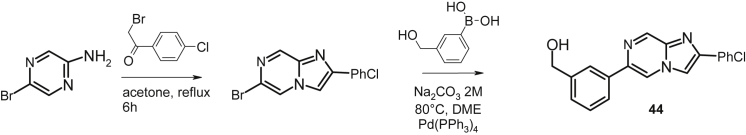
Synthesis of (3-(2-(4-chlorophenyl)imidazo[1,2-a]pyrazin-6-yl)phenyl)methanol (cpd 44).

**Scheme 12:**
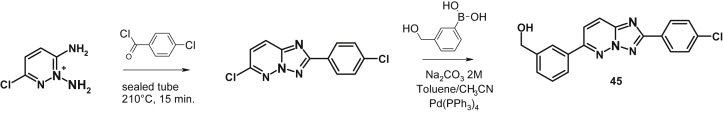
Synthesis of (3-(2-(4-chlorophenyl)-[1,2,4]triazolo[1,5-b]pyridazin-6-yl)phenyl)methanol (cpd 45).

**Scheme 13:**
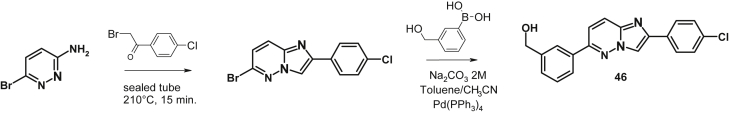
Synthesis of (3-(2-(4-chlorophenyl)imidazo[1,2-b]pyridazin-6-yl)phenyl)methanol (cpd 46).

**Scheme 14:**
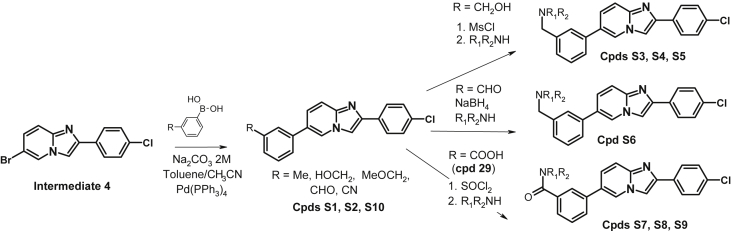
Synthesis of 2-(4-Chlorophenyl)-6-(m-tolyl)imidazo[1,2-a]pyridine (cpd S1).

**Scheme 15:**
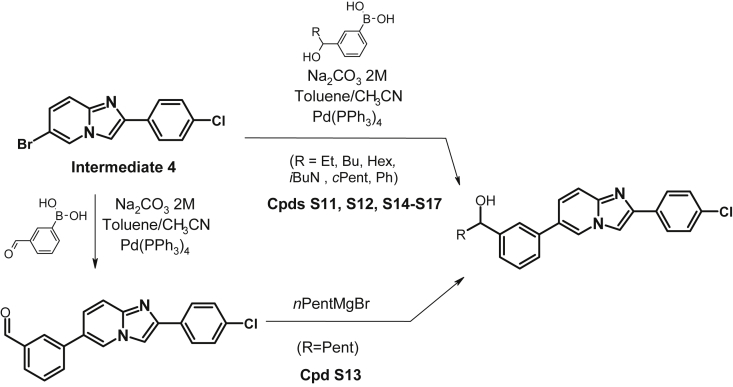
Synthesis of 1-(3-(2-(4-chlorophenyl)imidazo[1,2-a]pyridin-6-yl)phenyl)propan-1-ol (cpd S11), 1-(3-(2-(4-chlorophenyl)imidazo[1,2-a]pyridin-6-yl)phenyl)pentan-1-ol (cpd S12), 1-(3-(2-(4-chlorophenyl)imidazo[1,2-a]pyridin-6-yl)phenyl)hexan-1-ol (cpd S13), 1-(3-(2-(4-chlorophenyl)imidazo[1,2-a]pyridin-6-yl)phenyl)heptan-1-ol (cpd S14), 1-(3-(2-(4-chlorophenyl)imidazo[1,2-a]pyridin-6-yl)phenyl)-3-methylbutan-1-ol (cpd S15), (3-(2-(4-chlorophenyl)imidazo[1,2-a]pyridin-6-yl)phenyl)(cyclopentyl)methanol (cpd S16), (3-(2-(4-chlorophenyl)imidazo[1,2-a]pyridin-6-yl)phenyl)(phenyl)methanol (cpd S17).

**Fig. 1:**
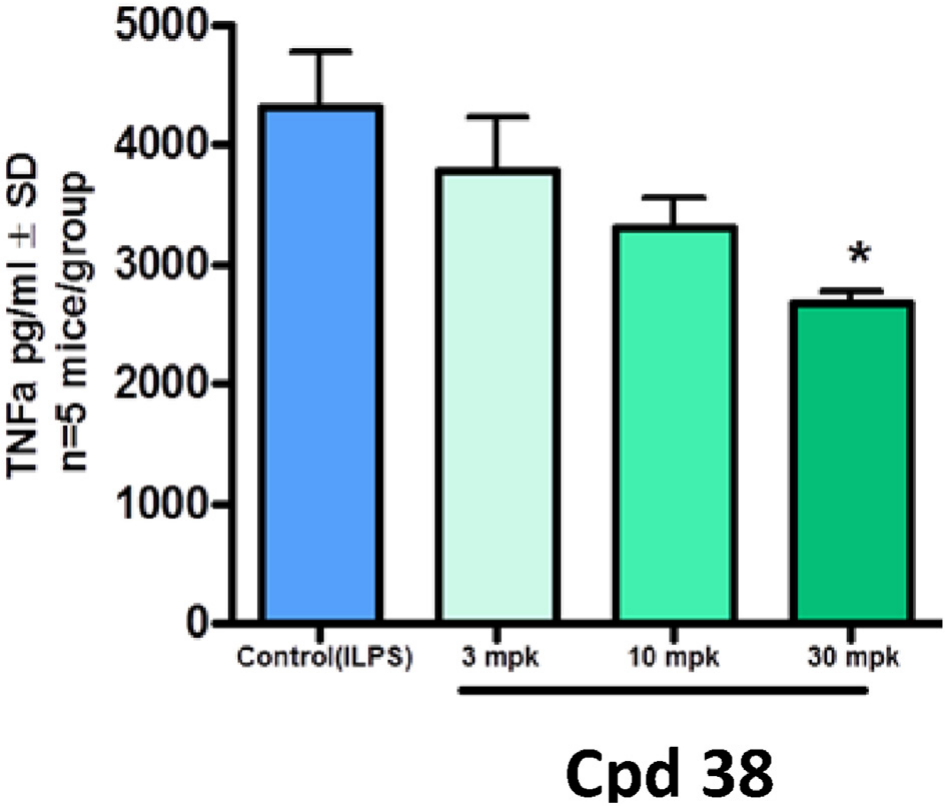
Serum TNF-alpha levels (mean) in mice.

The data shared in this article describes the steps involved in the optimization of a series of ligands of the NURR1/NOT nuclear receptor to yield a potent agonist (cpd 38) with promising anti-inflammatory and neuroprotective pharmacological activities and good druggability. The report contains the chemical schemes for the synthesis of all new compounds (Scheme 1, Scheme 2, Scheme 3, Scheme 4, Scheme 5, Scheme 6, Scheme 7, Scheme 8, Scheme 9, Scheme 10, Scheme 11, Scheme 12, Scheme 13, Scheme 14, Scheme 15) and the full procedure for the preparation of these compounds (cpds 1–46) along with all analytical descriptions and characterizations. Additional examples illustrating substitutions and modifications of the main lead compound 4 to afford compounds S1–S17 are included (Table 1, Table 2). Full procedures for evaluation of the compounds in all cellular assays, such as reporter gene assays, neuroprotection in dopaminergic neurons, cytokine assays in MG7 cells or neuroprotection in neurons-microglia cocultures are also described. In addition a full description of ADME assays (permeability, metabolism, clearance, CYP inhibition and induction is provided along with characterization of the most active compounds in these panels is provided. Full description of pharmacological evaluation in a neuroprotection model is also described to illustrate the activity of the most potent compounds as antiinflammatory and neuroprotective (Fig. 1). Finally the procedures for hemodynamic, electrocardiographic and electrophysiological evaluation of the most potent compounds in anesthetized mongrel dogs is described (Table 3, Table 4).

**Table 1:** This table illustrates that replacements for the hydroxymethyl of cpd 4 by a variety of alcoxy- or alkylamino-methyl, amides (with the exception of primary methyl amide) or cyano groups led to total loss of activity showing a stringent need for a hydrogen bond donor group at this position.

Cpd	R	EC_50_ Gal4	Emax Gal4
S1	Me	>3000	
S2	CH2OMe	>3000	
S3	CH2NMe2	>3000	
S4	CH2pyrrolidino	>3000	
S5	CH2morpholino	>3000	
S6	CH2NHMe	>3000	
S7	CONH2	1585 ± 35	210 ± 5
S8	CONHMe	87 ± 28	226 ± 66
S9	CONEt2	>3000	
S10	CN	>3000

**Table 2:** This table illustrates that replacements for the methyl of **cpd 4** by a variety of alkyl or aryl groups led to total loss of activity showing no toleration for any substituent larger than a methyl group.

Cpd	R	EC50 Gal4 t
S11	Et	>1000
S12	Bu	>3000
S13	pent	>3000
S14	hex	>3000
S15	iBu	>3000
S16	cyclopent	>3000
S17	Ph	>3000

**Table 3:** - Plasma and heart concentrations of Cpd 38 after 5-min i.v. administration in anesthetized mongrel dogs (values are mean ± SD with n = 3 per group).

Dose	Sampling time	Total plasma concentration		Heart concentration	
mg/kg	min	μg/mL	μM	μg/g	μM
10	10	23.4 ± 2.83	64.5	Not taken	Not taken
	60	10.1 ± 0.769	27.8	14.6 ± 2.70	40.2

**Table 4:** Plasma and heart concentrations of Cpd 38 after 5-min i.v. administration in anesthetized male Hartley guinea pigs (values are mean ± SD with n = 6 per group).

Dose	Sampling time	Total plasma concentration		Heart concentration	
mg/kg	min	μg/mL	μM	μg/g	μM
10	10	4.66 ± 1.35	12.8	Not taken	Not taken
	30	2.57 ± 0.533	7.1	6.01 ± 0.964	16.6

## 2 Experimental design, materials, and methods

### 2.1 Chemistry

For the sake of clarity, compounds numbering will be kept the same as in the related research article referenced above [1]. A number of these compounds syntheses have been described in patents and will not be reported here. However, for the sake of clarity the chemical synthesis schemes will be kept in the present article.

The synthesis of the two lead compounds 3 and 4 is straightforward using classical chemistry reported for synthesis of imidazo[1,2a]pyridines [3] (Scheme 1).

#### 2.1.1 N-Phenyl-6-(pyrid-2-yl)imidazo[1,2-a]pyridine-2-carboxamide (cpd 3) [4]

.

#### 2.1.2 (3-[2-(4-Chlorophenyl)imidazo [1, 2-a]pyridin-6-yl]phenyl)methanol (cpd 4) [6]

.

#### 2.1.3 6-(pyrid-2-yl)imidazo[1,2-a]pyridine-2-carboxylic acid (cpd 11) [7]

.

#### 2.1.4 Preparation of analogs of cpd 3 varying by the amide substitution (compounds 12–28)

The compounds 12 to 28 are obtained by coupling the 6-(pyridin-2-yl)imidazo[1,2-a]pyridine-2-carboxylic acid with the appropriate aromatic amines according to the procedures described in Refs. [4], [6].

**N-3,5-difluorophenyl-6-(pyrid-2-yl)imidazo[1,2-a]pyridine-2-carboxamide (cpd 12)** [5].

**N-2-chlorophenyl-6-(pyrid-2-yl)imidazo[1,2-a]pyridine-2-carboxamide (cpd 13)** [5].

**N-3-fluorophenyl-6-(pyrid-2-yl)imidazo[1,2-a]pyridine-2-carboxamide (cpd 14)** [5].

**N-2-fluorophenyl-6-(pyrid-2-yl)imidazo[1,2-a]pyridine-2-carboxamide (cpd 15)** [5].

**N-2,5-difluorophenyl-6-(pyrid-2-yl)imidazo[1,2-a]pyridine-2-carboxamide (cpd 16)** [5].

**N-2,3-difluorophenyl-6-(pyrid-2-yl)imidazo[1,2-a]pyridine-2-carboxamide (5, cpd 17)** [5].

**N-2-cyanophenyl-6-(pyrid-2-yl)imidazo[1,2-a]pyridine-2-carboxamide (cpd 18)** [5].

**N-6-di(pyridin-2-yl)imidazo[1,2-a]pyridine-2-carboxamide** (**cpd 19)** [7].

**N-6-fluoropyridin-2-yl-6-(pyrid-2-yl)imidazo[1,2-a]pyridine-2-carboxamide (cpd 20)** [7].

**N-Thien-3-yl -6-(pyrid-2-yl)imidazo[1,2-a]pyridine-2-carboxamide (cpd 21)** [7].

**N-Thiazol-2-yl -6-(pyrid-2-yl)imidazo[1,2-a]pyridine-2-carboxamide (cpd 22)** [7].

**N-Isoxazol-3-yl -6-(pyrid-2-yl)imidazo[1,2-a]pyridine-2-carboxamide (cpd 23)** [7].

**N-1,3,4-Thiazol-2-yl -6-(pyrid-2-yl)imidazo[1,2-a]pyridine-2-carboxamide (cpd 24)** [7].

**N-Pyrazol-3-yl -6-(pyrid-2-yl)imidazo[1,2-a]pyridine-2-carboxamide (cpd 25)** [7].

**N-Tetrahydropyridinyl -6-(pyrid-2-yl)imidazo[1,2-a]pyridine-2-carboxamide (cpd 27)** [8].

**N-Indolyl -6-(pyrid-2-yl)imidazo[1,2-a]pyridine-2-carboxamide (cpd 28)** [8].

#### 2.1.5 3-(2-(4-chlorophenyl)imidazo[1,2-a]pyridin-6-yl)benzoic acid (cpd 29)

**Figure:**
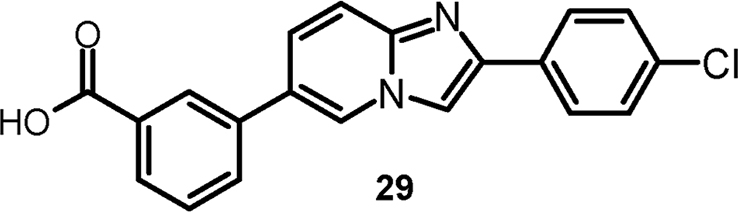

300 mg (0.98 mmol) of 6-bromo-2-(4-chlorophenyl)imidazo[1,2-a]pyridine (**Intermediate 2**, described above), 162 mg of 3-boronobenzoic acid and 56 mg of tetrakis(triphenylphosphine)palladium are mixed in a microwave tube containing 5 ml of acetonitrile and 5 ml of a 2M solution of sodium hydrogen carbonate. The tube is placed in a microwave apparatus and irradiated at 150′ C. for 15 min. The organic phase is separated, dried and concentrated under reduced pressure. A solid residue is obtained which is triturated in a mixture of 3 ml of dichloromethane and 3 ml of diisopropyl ether for 30 min. The precipitate is recovered by filtration, washed with diisopropyl ether and dried in a desiccator under reduced pressure. 342 mg of compound are obtained. ^1^H NMR (DMSO-d6, *δ* in ppm): 7.51 (d, J = 8.7Hz, 2H), 7.62–7.70 (m, 3H), 7.97–8.02 (m, 4H), 8.26 (t, J = 1.6Hz, 1H), 8.45 (s, 1H), 8.99 (s, 1H); LCMS (ESI): m/z 349 [M+H]+.

#### 2.1.6 {3-[2-(4-Chlorophenyl)imidazo[1,2-a]pyridin-6-yl]-2-fluorophenyl}-methanol (cpd 30) [9]

.

**Figure:**
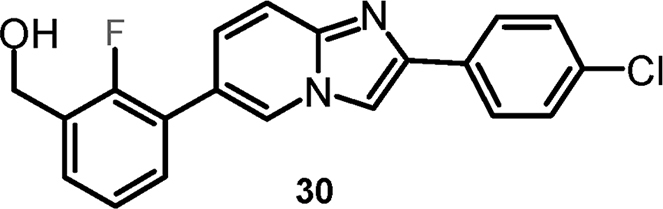

#### 2.1.7 {3-[2-(4-Chlorophenyl)imidazo[1,2-a]pyridin-6-yl]-2,6-difluorophenyl}methanol (cpd 31) [9]

.

**Figure:**
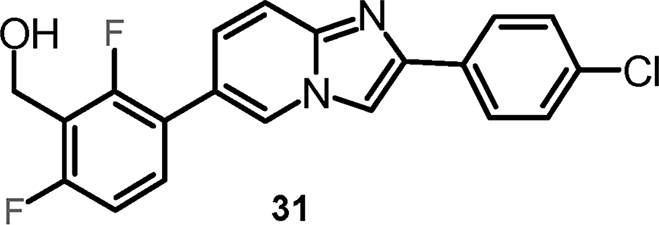

#### 2.1.8 {3-[2-(4-Chlorophenyl)imidazo[1,2-a]pyridin-6-yl]-6-fluorophenyl}-methanol (cpd 32) [9]

.

**Figure:**
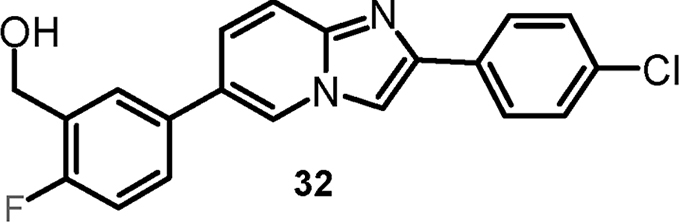

#### 2.1.9 {3-[2-(4-Chlorophenyl)imidazo[1,2-a]pyridin-6-yl]-6-methylphenyl}-methanol (cpd 33) [9]

.

**Figure:**
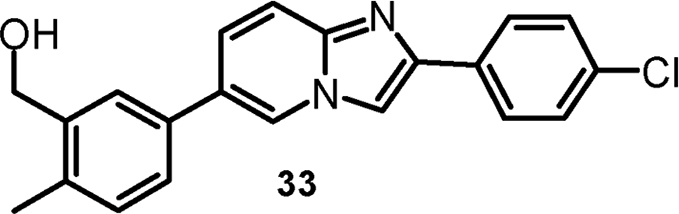

#### 2.1.10 {3-[2-(4-Chlorophenyl)imidazo[1,2-a]pyridin-6-yl]-2-methylphenyl}-methanol (cpd 34) [9]

.

**Figure:**
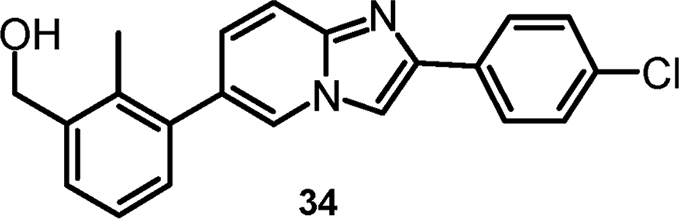

#### 2.1.11 1-(3-[2-(4-chlorophenyl)imidazo[1,2-a]pyridin-6-yl]phenyl)ethanol and enantiomers (cpds 35–37) [6]

.

**Figure:**
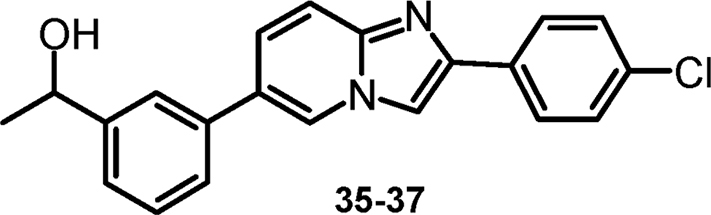

#### 2.1.12 2-(3-[2-(4-Chlorophenyl)imidazo[1,2-a]pyridin-6-yl]phenyl)propan-2-ol (cpd 38) [6]

.

#### 2.1.13 3-(3-(2-(4-chlorophenyl)imidazo[1,2-a]pyridin-6-yl)phenyl)oxetan-3-ol (cpd 39)

.

a. 2-(4-Chlorophenyl)-6-(4,4,5,5-tetramethyl-1.3.2-dioxaborolan-2-yl)imidazo[1,2-a]pyridine

5.0 g of 5-(4,4,5,5-tetramethyl-1,3,2-dioxaborolan-2-yl)pyridin-2-ylamine and 5.30 g of 2-bromo-1-(4-chlorophenyl)ethanone are placed in 150 ml of n-propanol in a round-bottomed flask. 2.67 g of sodium hydrogencarbonate are added. The mixture is heated at 80 °C. for 16 h. After cooling, the reaction mixture is concentrated under reduced pressure. 10.93 g of compound are obtained, which compoundis used as is in the following stages. ^1^H NMR (d6-DMSO, *δ* in ppm): 1.35 (s, 12H); 7.35 (d, 1H); from 7.5 to 7.6 (m, 3H); 7.95 (d, 2H); 8.45 (d, 1H); 7.85 (s, 1H); LCMS (ESI): m/z 355 [M+H]+.

b. 2-(4-Chlorophenyl)imidazo[1,2-a]pyridine-6-boronic acid hydro-chloride (1:1) (**Intermediate 4**)

7.93 g of 2-(4-chlorophenyl)-6-(4,4,5,5-tetramethyl-1,3,2-dioxaborolan-2-yl)imidazo[1,2-a]pyridine are dissolved in 200 ml of acetone and 100 ml of water; 223 ml of 1N hydrochloric acid are added thereto, dropwise there with stirring, and the mixture is stirred at ambient temperature for 16 h. The reaction mixture is subsequently concentrated under reduced pressure. 4.78 g of compound are obtained, which compound is used as is in the following stages. ^1^H NMR (d6-DMSO, *δ* in ppm): from 7.6 to 7.75 (m, 2H); 7.95 (m, 1H); from 8.05 to 8.15 (m, 2H); 8.2 (m, 1H); 8.9 (s, 1H); 9.1 (s, 1H); LCMS (ESI): m/z 273 [M+H]+.

c. 3-(3-(2-(4-chlorophenyl)imidazo[1,2-a]pyridin-6-yl)phenyl)oxetan-3-ol (**cpd 39**)

4.5 ml of tetrahydrofuran, 0.5 ml of water and 1 g of cesium carbonate solution are introduced into a round-bottomed flask and degassed under a stream of argon. 200 mg of 2-(4-chlorophenyl)imidazo[1,2-a]pyridine-6-boronic acid (**Intermediate 4**), 180 mg of 3-(3-bromophenyl)oxetan-3-ol [10] and 55 mg of PdCl_2_(dppf)-CH_2_Cl_2_ (1-1) are added. The mixture is heated at 70 °C. for 20h and then allowed to retum to ambient temperature. The solvent is evaporated under reduced pressure. It is taken up between ethyl acetate and water. The organic phase is separated and dried. The solvent is concentrated under reduced pressure. The residue is purified by chromatography on silica gel, elution being carried out with a dichloromethane/acetone mixture. 125 mg of compound are obtained. Mp = 180–182 °C.; ^1^H NMR (d6-DMSO, *δ* in ppm): 4.82 (d, J = 6.4Hz, 2H), 4.84 (d, J = 6.4Hz, 2H), 6.45 (s, 1H), 7.53 (d, J = 8.5Hz, 2H), 7.57 (d, J = 7.6Hz, 1H), 7.64–7.72 (m, 4H), 7.93 (t, J = 1.8Hz, 1H), 8.03 (d, J = 8.5Hz, 2H), 8.47 (s, 1H), 8.93 (m, 1H); LCMS (ESI): m/z 379 [M+H]+.

#### 2.1.14 4-(3-(2-(4-chlorophenyl)imidazo[1,2-a]pyridin-6-yl)phenyl)tetrahydro-2H-pyran-4-ol (cpd 40)

2.8 ml of ethanol, 8.2 ml of toluene and 1.94 ml of a 2M sodium carbonate solution are introduced into a round-bottomed flask and degassed under a stream of argon. 310 mg of 2-(4-chlorophenyl)imidazo[1,2-a]pyridine-6-boronic acid (**Intermediate 4**), 200 mg of 4-(3-bromophenyl) tetrahydro-2H-pyran-4-ol^v^ and 54 mg of tetrakis(triphenylphosphine)palladium are added. The mixture is heated at 80 °C. for 3h and then allowed to retum to ambient temperature. The solvent is evaporated under reduced pressure. It is taken up between ethyl acetate and water. The organic phase is separated and dried. The solvent is concentrated under reduced pressure. The residue is purified by chromatography on silica gel, elution being carried out with a dichloromethane/acetone mixture. 250 mg of compound are obtained. Mp = 224–225 °C**.;** ^1^H NMR (d6-DMSO, *δ* in ppm): 1.60 (broad d, J = 13.0Hz, 2H), 2.08 (td, J = 13.0, 13.0, 5.1Hz, 2H), 3.75 (dd, J = 10.9, 5.1Hz, 2H), 3.83 (td, J = 10.9, 10.9, 1.3Hz, 2H), 5.12 (s, 1H), 7.45–7.56 (m, 4H), 7.59 (dt, J = 7.6, 1.3, 1.3Hz, 1H), 7.64 (dd, J = 9.5, 1.5Hz, 1H), 7.68 (d, J = 9.5Hz, 1H), 7.83 (t, J = 1.5, 1H), 8.01 (d, J = 8.6Hz, 2H), 8.44 (s, 1H), 8.90 (t, J = 1.2Hz, 1H); LCMS (ESI): m/z 405 [M+H]+.

#### 2.1.15 2-(3-(2-(4-chlorophenyl)imidazo[1,2-a]pyridin-6-yl)phenyl)propane-1,2-diol (cpd 41)

35 ml of ethanol, 65 ml of toluene and 15.2 ml of a 2M sodium carbonate solution are introduced into a round-bottomed flask and degassed under a stream of argon. 2.43 g of 2-(4-chlorophenyl)imidazo[1,2-a ]pyridine-6-boronic acid (**Intermediate 4**), 1.4 g of 2-(3-bromophenyl)propane-1,2-diol and 350 mg of tetrakis(triphenylphosphine)palladium are added. The mixture is heated at 80 °C. for 3h and then allowed to retum to ambient temperature. It is taken up between ethyl acetate and water. The organic phase is separated and dried. The solvent is concentrated under reduced pressure. The residue is purified by chromatography on silica gel, elution being carried out with a dichloromethane/acetone/methanol mixture. 1.42 g of compound are obtained. Mp = 192–194 °C.; ^1^H NMR (DMSO-d6, *δ* in ppm): 1,47 (s, 3H); 3,50 (d, 2H); 4,70 (t, 1H); 5,06 (s, 1H); 7,4–7,69 (rn, 7H); 7,82 (s, 1H); 8,01 (d, J = 8,5Hz, 1H); 8,42 (s, 1H); 8,9 (s, 1H); LCMS (ESI): m/z 379 [M+H]+.

#### 2.1.16 2-{ 3-[2-(4-Chlorophenyl)imidazo[1,2-a]pyridin-6-yl]-2,6-difluoro-phenyl}propan-2-ol (cpd 42) [9]

.

#### 2.1.17 (3-(2-(4-chlorophenyl)indolizin-6-yl)phenyl)methanol (cpd 43)

a. 6-bromo-2-(4-chlorophenyl)indolizine

A solution of 5-Bromo-2-methylpyridine (2.93 mmol, 500 mg) and 2-bromo-4-chloroacetophenone (2.93 mol, 682 mg) in acetone (15 mL) was heated at reflux for 6 h. The quaternary salt was isolated via filtration and re-dissolved in hot (60–80 °C) water (10 mL). K2CO3 (2.93 mmol, 400 mg) was added and the mixture heated at 80 °C for 8 h. After filtration and drying in vacuo, 6-bromo-2-(4-chlorophenyl)indolizine (407 mg, 50%) was obtained. LCMS (ESI): m/z 305 [M+H]+.

b. (3-(2-(4-chlorophenyl)indolizin-6-yl)phenyl)methanol (**cpd 43**)

8 ml of dimethoxyethane and 3 ml of a 2M sodium carbonate solution are introduced into a round-bottomed flask and degassed under a stream of argon. 229 mg of 3-hydroxymethylbenzeneboronic acid, 370 mg of 6-bromo-2-(4-chlorophenyl)indolizine and 69 mg of tetrakis(triphenylphosphine)palladium are added. The mixture is heated at 80 °C. for 6h and then allowed to return to ambient temperature. It is taken up between ethyl acetate and water. The organic phase is separated and dried. The solvent is concentrated under reduced pressure. The residue is purified by chromatography on silica gel, elution being carried out with a dichloromethane/ethyl acetate mixture. 226 mg of compound are obtained. Mp = 177–179 °C.; ^1^H NMR (DMSO-d6, *δ* in ppm): 4.60 (d, J = 5.6Hz, 2H), 5.25 (t, J = 5.6Hz, 1H), 6.84 (s, 1H), 7.09 (dd, J = 9.3, 1.2Hz, 1H), 7.33 (d, J = 7.3Hz, 1H), 7.42–7.50 (m, 3H), 7.52 (d, J = 9.4Hz, 1H), 7.56 (d, J = 7.5Hz, 1H), 7.64 (t, J = 1.5Hz, 1H), 7.75 (d, J = 8.4Hz, 2H), 8.06 (d, J = 1.0Hz, 1H), 8.60 (s, 1H); LCMS (ESI): m/z 334 [M+H]+.

#### 2.1.18 (3-(2-(4-chlorophenyl)imidazo[1,2-a]pyrazin-6-yl)phenyl)methanol (cpd 44)

a. 6-bromo-2-(4-chlorophenyl)imidazo[1,2-a]pyrazine

A solution of 2-amino-5-bromopyrazine (500 mg, 2.87 mmol) in dimethoxyethane (10 ml) was charged into a round-bottomed flask, degassed under a stream of argon. 2-bromo-4′-chloroacetophenone (805 mg, 3.45 mmol) was added and the reaction mixture was stirred at room temperature for 20 hours and concentrated under reduced pressure. The residue was taken up with ethanol and heated at 100 °C. for 2 h 30. After cooling to ambient temperature, the residue was concentrated under reduced pressure and purified by chromatography on silica gel, elution being carried out with a heptane/ethyl acetate mixture. 60 mg of compound are obtained and used as such in the next step.

b. (3-(2-(4-chlorophenyl)imidazo[1,2-a]pyrazin-6-yl)phenyl)methanol (**cpd 44**)

A mixture of 253 mg (0.77 mmol) of cesium carbonate, 5 mL of dioxane, water (0.4 ml), 60 mg (0.19 mmol) of 6-bromo-2-(4-chlorophenyl)imidazo[1,2-a]pyrazine, 7 mg (0.05eq.) of [1,1′-bis(dipheny1phosphino)ferrocene]dichloropalladium and 59 mg (0.4 mmol) of 3-(hydroxymethyl)phenylboronic acid is heated for 3 hours at 130 °C. and then partially concentrated, diluted with dichloromethane and filtered. The organic phase is washed with water and dried over magnesium sulfate, filtered and concentrated to dryness under reduced pressure. The residue is chromatographed on a silica cartridge, eluting with a mixture of dichloromethane and methanol (90/10). The fractions containing the expected product are combined and concentrated to dryness under reduced pressure to give 32 mg of (3-(2-(4-chlorophenyl)imidazo[1,2-a]pyrazin-6-yl)phenyl)methanol. ^1^H NMR (d6-DMSO, *δ* in ppm): 4.60 (d, J = 5.4Hz, 2H), 5.28 (t, J = 5.4Hz, 1H), 7.37 (d, J = 7.4Hz, 1H), 7.47 (t, J = 7.7Hz, 1H), 7.56 (d, J = 8.6Hz, 2H), 7.92, (dt, J = 7.8, 1.6, 1.6Hz, 1H), 8.04 (t, J = 1.5Hz, 1H), 8.09 (d, J = 8.6Hz, 2H), 8.61 (s, 1H), 9.17 (s, 2H); LCMS (ESI): m/z 336 [M+H]+.

#### 2.1.19 (3-(2-(4-chlorophenyl)- [1], [2], [4]triazolo[1,5-b]pyridazin-6-yl)phenyl)methanol (cpd 45)

a. 6-Chloro-2-(4-chlorophenyl)-s-triazolo[1,5-b]pyridazine

200 mg (0.58 mmol) of N-aminodiazinium [6] and 4-chlorobenzoyl chloride (7.80 mol, 1.36 g, 1 ml) was heated in a sealed tube at 210 °C for 15 min. The reaction mixture was made alkaline with 30% of ammoniac hydroxide, diluted with 5 ml of water stirred for 1 h at room temperature. The precipitate was filtered off and the filtrate was extracted three times with dichloromethane. The extract was washed with water, dried over magnesium sulfate and concentrated. 30 mg of the title compound was obtained. Mp = 217-19 °C.; LCMS (ESI): m/z 266 [M+H]+.

b. (3-(2-(4-chlorophenyl)- [1], [2], [4]triazolo[1,5-b]pyridazin-6-yl)phenyl)methanol (**cpd 45**)

30 mg (0.11 mmol) of 6-Chloro-2-(4-chlorophenyl)-s-triazolo[1,5-b]pyridazine, 26 mg of 3-hydroxymethylbenzeneboronic acid and 4 mg of tetrakis(triphenylphosphine)palladium are mixed in a microwave tube containing 2 ml of acetonitrile, 2 ml of toluene and 2 ml of a 2M solution of sodium carbonate. The tube is placed in a microwave apparatus and irradiated at 150 °C. for 15 min. The organic phase is separated, dried and concentrated under reduced pressure. A solid residue is obtained which is triturated with dichloromethane. The precipitate is recovered by filtration, washed with diisopropyl ether and dried in a desiccator under reduced pressure. 16 mg of compound are obtained. Mp = 217–219 °C.; ^1^H NMR (d6-DMSO, *δ* in ppm): 4.64 (d, J = 5.7Hz, 2H), 5.35 (t, J = 5.7Hz, 1H), 7.49–7.59 (m, 2H), 7.65 (d, J = 8.5Hz, 2H), 8.02 (m, 1H), 8.13 (br s, 1H), 8.21–834 (m, 3H), 8.53 (d, J = 9.4Hz, 1H); LCMS (ESI): m/z 337 [M+H]+.

#### 2.1.20 (3-(2-(4-chlorophenyl)imidazo[1,2-b]pyridazin-6-yl)phenyl)methanol (cpd 46)

a. 6-bromo-2-(4-chlorophenyl)imidazopyridazine

A solution of 3-amino-6-bromopyridazine (2.87 mmol, 500 mg) and 2-bromo-4-chloroacetophenone (2.87 mmol, 670 mg) in n-propanol (25 mL) was heated at 90 °C for 20 h, and then allowed to return to ambient temperature. It is taken up between dichloromethane and water. The organic phase is separated and dried. The solvent is concentrated under reduced pressure. The residue is purified by recrystallization from isopropylether to give 569 mg of compound are obtained. LCMS (ESI): m/z 308 [M+H]+.

b. (3-(2-(4-chlorophenyl)imidazo[1,2-b]pyridazin-6-yl)phenyl)methanol (**cpd 46**)

300 mg (0.97 mmol) of 6-bromo-2-(4-chlorophenyl)imidazopyridazine, 221 mg of 3-hydroxymethylbenzeneboronic acid and 56 mg of tetrakis(triphenylphosphine) palladium are mixed in a microwave tube containing 5 ml of acetonitrile, 5 ml of toluene and 1.46 ml of a 2M solution of sodium carbonate. The tube is placed in a microwave apparatus and irradiated at 150 °C. for 15 min. The reaction mixture is filtered off, the organic phase is washed with water, dried and concentrated under reduced pressure. A solid residue is obtained which is triturated with dichloromethane. The precipitate is recovered by filtration, washed with diisopropyl ether and dried in a desiccator under reduced pressure. 240 mg of compound are obtained. Mp = 187–189 °C; ^1^H NMR (d6-DMSO, *δ* in ppm): 4.63 (d, J = 5.9Hz, 2H), 5.32 (t, J = 5.9Hz, 1H), 7.48–7.59 (m, 4H), 7.82 (d, J = 9.5Hz, 1H), 7.94 (dt, J = 6.5, 1.8, 1.8Hz, 1H), 8.02–8.13 (m, 3H), 8.22 (dd, J = 9.5, 0.5Hz, 1H), 8.96 (s, 1H); LCMS (ESI): m/z 336 [M+H]+.

The Compounds S1-S10 (Table 1) were synthesized from **Intermediate 4** using classical organic chemistry as depicted in the short descriptions below:

#### 2.1.21 2-(4-Chlorophenyl)-6-(m-tolyl)imidazo[1,2-a]pyridine (cpd S1)

Under a stream of nitrogen, 200 mg of 6-bromo-2-(4-chlorophenyl)imidazo[1,2-a]pyridine, 88 mg of 3-methylphenylboronic acid and 38 mg of dichlorobis(triphenylphosphine)palladium(II) are placed in a microwave tube containing 5 ml of acetonitrile, 5 ml of toluene and 6 ml of a 2M sodium carbonate solution degassed beforehand under a stream of nitrogen. The tube is placed in a microwave apparatus and irradiated at 150 °C. for 15 min. The organic phase is separated and dried and the filtrate is concentrated under reduced pressure. It is taken up between ethyl acetate and water. The organic phase is separated and dried. The solvent is concentrated under reduced pressure. The residue is purified by chromatography on silica gel, elution being carried out with a dichloromethane/acetone mixture. 126 mg of cpd 30 are obtained. Mp = 145–147 °C. ^1^H NMR (d6-DMSO, *δ* in ppm): 2.38 (s, 3H), 7.20 (d, J = 7.4Hz, 1H), 7.37 (t, J = 7.5Hz, 1H), 7.44–7.56 (m, 4H), 7.57–7.67 (m, 2H), 7.98 (d, J = 8.6Hz, 2H), 8.40 (s, 1H), 8.84 (dd, J = 1.5, 1.0Hz, 1H); LCMS (ESI): m/z 319 [M+H]+.

#### 2.1.22 2-(4-Chlorophenyl)-6-(3-(methoxymethyl)phenyl)imidazo[1,2-a]pyridine (cpd S2)

By working in the same manner as for the preparation of the cpd 30, replacing the 3-methylphenylboronic acid with 3-(methoxymethyl)phenylboronic acid. 70 mg of 2-(4-Chlorophenyl)-6-(3-(methoxymethyl)phenyl)imidazo[1,2-a]pyridine (cpd 31) is obtained. ^1^H NMR (d6-DMSO, *δ* in ppm): 3.35 (s, 3H), 4.51 (s, 2H), 7.35 (br d, J = 7.6Hz, 1H), 7.44–7.72 (m, 4H), 8.01 (d, J = 8.6Hz, 2H), 8.44 (s, 1H), 8.90 (dd, J = 1.5, 1.0Hz, 1H); MP: 135–137 °C; LCMS (ESI): m/z 349 [M+H]+.

#### 2.1.23 1-(3-(2-(4-chlorophenyl)imidazo[1,2-a]pyridin-6-yl)phenyl)-N,N-dimethylmethanamine (cpd S3)

a. (2.16 g, 6.06 mmol) of (3-[2-(4-Chlorophenyl)imidazo [1,2-a]pyridin-6-yl]phenyl)methanol (**cpd 4**) was dissolved in anhydrous THF and cooled to 0 °C. Triethylamine (0.92 g, 9.10 mmol) was added dropwise. After stirring for 10 min, methanesulfonyl chloride (0.76 g, 6.67 mmol) was added dropwise. The reaction mixture was stirred at 0 °C for 2 hours. At this time, more triethylamine (3.8 ml, 4.5eq.) and more methanesulfonyl chloride (1.36 ml, 3.eq) were added. After stirring at 0 °C for 1 hour, the solvent was removed, and the residue taken up between ethyl acetate and water. The organic phase is separated and dried. The solvent is concentrated under reduced pressure. The residue was purified by flash chromatography on silica gel eluting with 2% methanol in dichloromethane. 788 mg of the title product are obtained and used without further purification.
b. 1-(3-(2-(4-chlorophenyl)imidazo[1,2-a]pyridin-6-yl)phenyl)-N,N-dimethylmethanamine (**Cpd S3**): 3-(2-(4-chlorophenyl)imidazo[1,2-a]pyridin-6-yl)benzyl methanesulfonate (175 mg, 0.42 mmol) was treated with an dimethylamine (40% in water) (10eq.) in DMF (7 ml) at room temperature for 20 h. The solvent was removed in vacuo and the resulting residue was taken up between ethyl acetate and water. The organic phase is washed twice with water. The solvent is concentrated under reduced pressure and dried. 67 mg of the title cpd was obtained^.^ Mp = 290-92 °C.; ^1^H NMR (DMSO-d6, *δ* in ppm, hydrochloride salt): 2.75 (d, J = 4.8Hz, 6H), 4.37 (d, J = 5.2Hz, 2H), 7.59–7.72 (m, 4H), 7.81–7.89 (m, 2H), 8.02–8.13 (m, 4H), 8.67 (s, 1H), 9.16 (s, 1H), 10.86 (br, 1H); LCMS (ESI): m/z 362 [M+H]+.

#### 2.1.24 1-(3-(2-(4-chlorophenyl)imidazo[1,2-a]pyridin-6-yl)phenyl)-N-pyrolidine (cpd S4)

3-(2-(4-chlorophenyl)imidazo[1,2-a]pyridin-6-yl)benzyl methanesulfonate (175 mg, 0.42 mmol) was treated with pyrrolidine (10eq.) in DMF (7 ml) at room temperature for 20 h. The solvent was removed in vacuo and the resulting residue was taken up between ethyl acetate and water. The organic phase is washed twice with water. The solvent is concentrated under reduced pressure and dried. 69 mg of the title cpd was obtained. Mp = 348-50 °C.; ^1^H NMR (DMSO-d6, *δ* in ppm, hydrochloride salt): 1.98 (m, 4H), 3.11 (m, 2H), 3.39 (m, 2H), 4.44 (d, J = 5.4Hz, 2H), 7.55–7.71 (m, 4H), 7.86 (dt, J = 7.0, 1.6, 1.6Hz, 1H), 7.96 (d, J = 9.0Hz, 1H), 8.03–8.23 (m, 4H), 8.70 (s, 1H), 9.21 (br s, 1H), 11.25 (br, 1H); LCMS (ESI): m/z 388 [M+H]+.

#### 2.1.25 1-(3-(2-(4-chlorophenyl)imidazo[1,2-a]pyridin-6-yl)phenyl)-N-morpholine (cpd S5)

3-(2-(4-chlorophenyl)imidazo[1,2-a]pyridin-6-yl)benzyl methanesulfonate (175 mg, 0.42 mmol) was treated with morpholine (10eq.) in DMF (7 ml) at room temperature for 20 h. The solvent was removed in vacuo and the resulting residue was taken up between ethyl acetate and water. The organic phase is washed twice with water. The solvent is concentrated under reduced pressure and dried. 42 mg of the title cpd was obtained. Mp = 348-50 °C.; ^1^H NMR (DMSO-d6, *δ* in ppm, hydrochloride salt): 3.00–3.33 (m, 4H), 3.75–4.00 (m, 4H), 4.41 (d, J = 3.7Hz, 2H), 7.56–7.69 (m, 4H), 7.85 (dt, J = 6.2, 2.0, 2.0Hz, 1H), 7.95 (d, J = 9.4Hz, 1H), 8.00–8.20 (m, 4H), 8.68 (s, 1H), 9.18 (s, 1H), 11.60 (br, 1H); LCMS (ESI): m/z 404 [M+H]+.

#### 2.1.26 1-(3-(2-(4-chlorophenyl)imidazo[1,2-a]pyridin-6-yl)phenyl)-N-methylmethanamine (cpd S6)

a. 3-(2-(4-chlorophenyl)imidazo[1,2-a]pyridin-6-yl)benzaldehyde

500 mg of 6-bromo-2-(4-chlorophenyl)imidazo[1, 2-a]pyridine (described above), 243 mg of (3-formylphenyl)boronic acid and 94 mg of tetrakis(triphenylphosphine)palladium are placed under a stream of argon in a in a microwave tube comprising a mixture, degassed beforehand under a stream of argon, of 5 ml of acetonitrile, 5 ml of toluene and 6ml of a 2M sodium carbonate solution. The tube is placed in a microwave apparatus and irradiated at 150 °C. for 15 min. After cooling, the reaction mixture is diluted with ethyl acetate and water and then the organic phase is separated, dried and evaporated under reduced pressure. The residue is purified by chromatography on silica gel, elution being carried out with a dichloromethane/MeOH 99/01 mixture. 380 mg of cpd are obtained. Mp = 124–126 °C.; LCMS (ESI): m/z 333 [M+H]+.

b. To a solution of 3-(2-(4-chlorophenyl)imidazo[1,2-a]pyridin-6-yl)benzaldehyde (200 mg, 0.6 mmol, 1.0 equiv) in methanol (10 ml) was added was added a 2N solution of methylamine (0.38 ml, 0.77 mmol, 1.3 equiv) in methanol. The reaction mixture was stirred at room temperature for 15 min. After cooling to 0 °C, sodium borohydride (11.5 mg, 0.3 mmol, 0.5 equiv) was added portion-wise. The reaction mixture was stirred for 3 h at room temperature and solvents were removed in vacuo. The reaction was quenched very slowly by the addition of aqueous 2N NaOH (20 ml). Ethyl acetate was added and the layers were separated. The organic layer was washed with brine, dried (MgSO4), filtered and concentrated under reduced pressure. The compound was purified by column chromatography (eluted with 5% methanol in dichloromethane) to afford 74 mg of desired compound. Mp = 322–324 °C; LCMS (ESI): m/z 348 [M+H]+.

#### 2.1.27 3-(2-(4-chlorophenyl)imidazo[1,2-a]pyridin-6-yl)benzamide (cpd S7)

In a 50 mL two-necked round-bottom flask equipped with a drying tube was added 150 mg (0.43 mmol) of 3-(2-(4-chlorophenyl)imidazo[1,2-a]pyridin-6-yl)benzoic acid (cpd 29) and dichloroethane (2 ml). Thionyl chloride (200 mg, 1.72 mmol) was then added, dropwise, over 5 min. The mixture was stirred under warm conditions about 85 °C and refluxed for 2 h until hydrogen chloride gas was no longer evolved. The precipitated white solid was filtered off, washed with isopropylether and dry. To the acyl chloride obtained, dissolved in dichloromethane (2 ml) was added in one portion of 1ml of a 7N solution of amoniac in methanolnol at room temperature. The reaction mixture was stirred for 20 hours at room temperature and then was diluted with dichloromethane. The solution was transferred to a separation funnel and was washed with 1 N HCl. The organic layer was dried with Na2SO4, filtered and concentrated under reduced pressure. Trituration of the resulting solid with hexane followed by filtration afforded the pure amide. Mp = 246–248 °C; LCMS (ESI): m/z 348 [M+H]+.

#### 2.1.28 3-(2-(4-chlorophenyl)imidazo[1,2-a]pyridin-6-yl)-N-methylbenzamide (cpd S8)

By working in the same manner as for the preparation of the cpd 38, replacing the diethylamine with 1ml of an 8N solution of methylamine in ethanol. 63 mg of 3-(2-(4-chlorophenyl)imidazo[1,2-a]pyridin-6-yl)-N-methylbenzamide is obtained; Mp = 225–227 °C; ^1^H NMR (d6-DMSO, *δ* in ppm): 2.84 (d, J = 4.6Hz, 3H), 7.51 (d, J = 8.5Hz, 2H), 7.60 (t, J = 7.7Hz, 1H), 7.66–7.72 (m, 2H), 7.81–7.92 (m, 2H), 8.02 (d, J = 8.5Hz, 2H), 8.17 (t, J = 1.5Hz, 1H), 8.45 (s, 1H), 8.55 (q, J = 4.6Hz, 1H), 8.96 (t, J = 1.4Hz, 1H); LCMS (ESI): m/z 362 [M+H]+.

#### 2.1.29 3-(2-(4-chlorophenyl)imidazo[1,2-a]pyridin-6-yl)-N,N-diethylbenzamide (cpd S9)

In a 50 mL two-necked round-bottom flask equipped with a drying tube was added 150 mg (0.43 mmol) of 3-(2-(4-chlorophenyl)imidazo[1,2-a]pyridin-6-yl)benzoic acid (**cpd 29**) and dichloroethane (2 ml). Thionyl chloride (200 mg, 1.72 mmol) was then added, dropwise, over 5 min. The mixture was stirred under warm conditions about 85 °C and refluxed for 2 hours until hydrogen chloride gas was no longer evolved. The precipitated white solid was filtered off, washed with isopropylether and dry. To the acyl chloride obtained, dissolved in dichloromethane (2 ml) was added in one portion a solution of diethylamine (0.5 ml) in ethanol (2 ml) at room temperature. The reaction mixture was stirred for 20 hours at room temperature and then was diluted with dichloromethane. The solution was transferred to a separation funnel and was washed with 1 N HCl. The organic layer was dried with Na2SO4, filtered and concentrated under reduced pressure. Trituration of the resulting solid with hexane followed by filtration afforded the pure amide. Mp = 162–164 °C; ^1^H NMR (d6-DMSO, *δ* in ppm): 1.15 (br, 6H), 3.45 (br, 4H), 7.36 (dt, J = 7.5, 1.3, 1.3Hz, 1H), 7.46–7.62 (m, 3H), 7.64–7.72 (m, 3H), 7.80 (dt, J = 7.8, 1.5, 1.5Hz, 1H), 8.01 (d, J = 8.4Hz, 2H), 8.42 (s, 1H), 8.97 (t, J = 1.4Hz, 1H); LCMS (ESI): m/z 404 [M+H]+.

#### 2.1.30 3-(2-(4-chlorophenyl)imidazo[1,2-a]pyridin-6-yl)benzonitrile (cpd S10)

By working in the same manner as for the preparation of the cpd 30, replacing the 3-methylphenylboronic acid with 3-cyanophenylboronic acid. 57 mg of 3-(2-(4-chlorophenyl)imidazo[1,2-a]pyridin-6-yl)benzonitrile is obtained; Mp = 195–197 °C; ^1^H NMR (d6-DMSO, *δ* in ppm): 7.52 (d, J = 8.6Hz, 2H), 7.66–7.77 (m, 3H), 7.87 (dt, J = 7.7, 1.3, 1.3Hz, 1H), 8.03 (d, J = 8.6Hz, 2H), 8.10 (ddd, J = 7.8, 1.9, 1.2Hz, 1H), 8.26 (t, J = 1.5Hz, 1H), 8.43 (s, 1H), 9.03 (t, J = 1.3Hz, 1H); LCMS (ESI): m/z 330 [M+H]+.

The Compounds S11-S17 (Table 2) were synthesized starting from Intermediate 4 using classical organic chemistry as depicted in the short descriptions below and fully described in Ref. [6]:

**1-(3-(2-(4-chlorophenyl)imidazo[1,2-a]pyridin-6-yl)phenyl)propan-1-ol (cpd S11).**

**1-(3-(2-(4-chlorophenyl)imidazo[1,2-a]pyridin-6-yl)phenyl)pentan-1-ol (cpd S12).**

**1-(3-(2-(4-chlorophenyl)imidazo[1,2-a]pyridin-6-yl)phenyl)hexan-1-ol (cpd S13).**

**1-(3-(2-(4-chlorophenyl)imidazo[1,2-a]pyridin-6-yl)phenyl)heptan-1-ol (cpd S14).**

**1-(3-(2-(4-chlorophenyl)imidazo[1,2-a]pyridin-6-yl)phenyl)-3-methylbutan-1-ol (cpd S15).**

**(3-(2-(4-chlorophenyl)imidazo[1,2-a]pyridin-6-yl)phenyl)(cyclopentyl)methanol (cpd S16).**

**(3-(2-(4-chlorophenyl)imidazo[1,2-a]pyridin-6-yl)phenyl)(phenyl)methanol (cpd S17).**

## 3 Reporter gene assays

### 3.1 N2A cell line

N2A cells obtained from ATCC were stably transfected with NBRE8x (5′-AAGGTCA-3′) in tandem coupled with luciferase reporter gene. Each NBRE sequence was separated with 5 nucleotides. N2A were grown in 75 cm^2^ flask in DMEM containing 10% calf serum, 4.5 g/L glucose and 0.4 mg/ml geneticin. After one week in culture cells were harvested by trypsinization (0.25%, 30 minutes) then plated onto 96 well dishes at the density of 60 000 cell per well in 75 μl of DMEM without phenol red containing 4.5 g/l glucose, 10% lipid free serum from Hyclone. After 24 hours compounds were added (25 μl/well) at different concentration for further 24 hours. Measurement is performed by adding 100 μl of Steadylite per well for 30 minutes and plates are read *using microplate fluorescent reader.*

### 3.2 *CHO* cell line

CHO cells from ATCC were stably transfected with NBRE3X in tandem coupled with luciferase. Each NBRE sequence was separated by 8 nucleotides. CHO-NBRE3X-luc cells were seeded at 50 000 cells per well in black 96 well microplates in DMEM/F12 supplemented with 10% FBS. After 24 hours cells were treated with compounds at indicated concentrations (from 10^−9^ M to 3 10^−6^ M), and were further incubated for 24 hours. The luciferase activity was analyzed in cell extract using Steady Glow from Promega. The luminescence was determined with Wallac MicroBeta Counter (PerkinElmer). The activity of compound is calculated by the percentage of luciferase activity in cells stimulated in the presence of tested compound versus control (DMSO).

## 4 Primary cultures of rat dopaminergic neurons

Primary culture of ventral midbrain neurons from fourteen-day-old rat embryos was maintained in DMEM/F12 supplemented with N2. Treatment with compound was performed at the same time than plating. After 4 days in culture, the number of TH-positive cells was evaluated after immunostaining with an anti-TH monoclonal antibody (6 wells/condition and 12 wells for control). Results are expressed as % of control.

## 5 Cytokine assay in MG7 cells

The microglial cell line MG7 (ATCC) was maintained in DMEM Glutamax-1 with 4.5 g/l glucose supplemented with 10% FCS and 1% Gentamycin. Cells are grown in 75 cm^2^ TPP flasks at 37 °C in a 95% humidified 5% CO2 tissue culture incubator.

For cytokine quantitation, MG7 cells were placed at 2.5 × 10^5^ cells/well in 96-well tissue culture plates and incubated with **cpd 38** for 1 hour before activation. After the pre-incubation with **cpd 38**, cells were treated with 10 ng/ml LPS (lipopolysaccharide, Salmonella typhimurium, Sigma).

After 20 hours incubation, supernatants were collected for cytokine measurement using specific ELISA (R&D Systems).

## 6 Neuroprotection assay in neurons-microglia co-culture

Primary neurons from E16 mouse embryos were cultured for 5 days before adding primary mouse microglia. After the addition of LPS (0.1 μg/ml) with or without compounds to the co-culture, neurotoxicity was evaluated after 48 hours incubation by the measurement of MAP2 staining using InCell Western (Odyssey).

## 7 ADME assays

### 7.1 Metabolic clearance

Intrinsic clearance in human and in pharmacological or toxicological species (expressed in μL/minute/mg proteins) is assessed, in standard conditions, by kinetics analysis (time points: 0, 5, 10, 20 and 30 minutes) using 1 μM of NCE and 0.5 mg proteins/mL of liver microsomes (pool of more than 100 donors), in presence of 1 mM NADPH. Incubation conditions with hepatic microsomal fractions are the following: Microsomal protein concentration: 0.5 mg/mL; Substrate concentration: 1 μM; DMSO (solvant) maximal concentration in incubation: 0.2%; Cofactor: 1 mM NADPH; Kinetics time points: 0, 5, 10, 20 and 30 minutes; CYP isoforms inhibitor: potent and specific inhibitor such as ketoconazole 3 μM or azamuline 1 μM for CYP3A or aminobenzotriazole 1 mM for all CYPs. Percentage of drug remaining is calculated for each compound by normalizing the data at different time point to the data at T0 for analyte/internal standard peak area ratio: % stability = (peak area ratio at Tx/peak area ratio at T0) *100. Elimination slope (-k) was determined using a linear regression of the neperian logarithm (ln) of the percent remaining drug versus incubation time. k is expressed in minute-1.Intrinsic clearance (Clint) calculation is Clint = k/protein concentration *1000; Protein concentration in mg/mL; Clint is expressed in μL/minute/mg proteins.

### 7.2 CACO-2/TC7 permeability assay

An in vitro model utilizing Caco-2 cells is performed in the apical to basolateral (A→B) direction with a pH gradient and a BSA gradient, conditions that most closely reflect the conditions in the *in vivo* situation. This condition is routinely used for primary screening using 20 μM for compound concentration. Asymmetrical conditions are used with standard apical medium (0.5% BSA at pH 6.5) and standard basal medium (5% BSA at pH 7.4). DMSO final concentration is 1% (v/v) and assay time is 2h, at 37 °C, under optimized agitation, without CO2. The apparent permeability (Papp) is calculated as follows:

$$P_{app}=\frac{R_{2}}{\left[D_{0}\right]\times S\times t}$$

where R2 is the receiver quantity of compound after 2 hours (corresponding peak area or calculated concentration), [D0] is the donor concentration of test solution (corresponding peak area or calculated concentration), S is the insert area: (0.08 cm2 for BD HTS 96-well insert system) and t is the time (2 hours ie 7200 seconds) recovery. Papp values above 20 are rated high.

### 7.3 CYP inhibition in human hepatic microsomal fractions

The competitive inhibitory potencies of the compounds (final concentrations: 0–0.012–0.042–0.12–0.36 –0.5 – 1 –2 – 3.5–5–10 –20 – 30 μM) in human hepatic microsomal fractions. Human hepatic microsomal fractions (final protein concentration = 0.05 mg/mL) are used with phosphate buffer 0.1 M, pH 7.4 and 1 mM NADPH as cofactor.

- For CYP3A4 probe substrates are midazolam (3 μM) or testosterone (50 μM) (near Km) with solvant concentration in incubation of DMSO 0.33% for midazolam and DMSO 0.3% + Methanol 0.5% for testosterone. Incubation times are 10 minutes at 37 °C. The reference inhibitor is Ketoconazole for CYP3A. Enzyme activity is stopped with 1 volume of acetonitrile containing internal standard.

Following protein precipitation with acetonitrile and their removal by centrifugation, supernatant fluids are analyzed by mass-spectrometry or equivalent analytical method for assessment of 1′-hydroxymidazolam and/or 6b-hydroxytestosterone metabolites formation.

- For CYP2C9 probe substrate is Diclofenac (5 μM) (near Km) with solvant concentration in incubation of DMSO 0.5%. Incubation time is 10 minutes at 37 °C. Sulfaphenazole is the reference inhibitor.

Enzyme activity is stopped with 1 volume of acetonitrile containing internal standard. Following protein precipitation with acetonitrile and their removal by centrifugation, supernatant fluids are analyzed by mass-spectrometry or equivalent analytical method for assessment of 4′-Hydroxydiclofenac.

- For CYP2D6 probe substrate is Dextromethorphan (5 μM) (near Km) with solvant concentration in incubation of DMSO 0.5%. Incubation time is 30 minutes at 37 °C. Quinidine is the reference inhibitor. Enzyme activity is stopped with 1 volume of acetonitrile containing internal standard. Following protein precipitation with acetonitrile and their removal by centrifugation, supernatant fluids are analyzed by mass-spectrometry or equivalent analytical method for assessment of Dextrorphan.

IC50-values are calculated using Biostat-Speed LTS tool; metabolite concentrations measured are plotted versus NCE initial concentrations, following the 4 parameters equation:

Y = a + (b / (1 + exp [- slope * (ln(x) – ln(c))]),

where a is the minimal activity (100% inhibition), fixed to 0, in most of cases, b is the maximal activity (0% inhibition), fixed to the mean of metabolite concentration measured in wells with vehicle (DMSO), in most of cases, slope is the Hill number, the slope of the curve at the inflexion point. IC50 are expressed in μM.

### 7.4 Cyp induction

Fresh and/or validated lots of cryo-preserved hepatocytes are used for these assays. After assessment of cellular viability using the trypan blue exclusion test, hepatocytes were seeded on collagen-coated plates. Hepatocytes are generally seeded at a density of 1.25–2 x 10^5^ cells/cm2 and cultured in seeding media for at least 4 hours. For induction, hepatocytes are treated daily with incubation medium during 48-hrs. Hepatocytes are treated with known inducers or test compounds for 48-hrs followed by total RNA isolation followed by RT-PCR analysis using specific Taqman probes or specific mRNA quantification with bDNA analysis using Affymetrix sets. The potential induction effect of the compounds was tested for human hepatocytes on CYP1A2, CYP2B6 and CYP3A4 isoforms. The reference inducers used for calculations of % of Emax are omeprazole at 50 μM for CYP1A2, phenobarbital at 1 mM for CYP2B6 and rifampicin at 10 μM for CYP3A4. The medium used was William Medium E with Glutamax, 1% ITS, 50 μg/mLGentamycin, 0.1 μM dexamethasone and 0.1% BSA. Hepatocytes are incubated with reference inducers or the compounds at 1–10 μM. The final DMSO concentration does not exceed 0.6%. Assay time is 48hrs, 37 °C and 5% CO2 in cell culture incubators.

Each CYP isoform result will be expressed as % of Emax. Emax being the effect observed with the corresponding reference inducer. The extent of induction, % of positive control, resulting from treatment with the tested compound will be calculated as follows:

$$\text{\%}\;\text{of}\;\text{Emax}=\frac{E_{\mathrm{compound}}-E_{\mathrm{vehicule}\;\mathrm{control}}}{E_{positive\;control}-E_{vehicle\;control}}\times 100\%$$

where Ecompound is the relative-fold mRNA expression with tested compound-treatment, Epositive control is the relative-fold mRNA expression with reference inducer-treatment and Evehicle control is the relative-fold mRNA expression with vehicle-treatment (DMSO).

The individual value of % of Emax (vs reference inducer) and Fold Induction (vs vehicle) are calculated and reported. For CYP3A4, if the highest individual value is ≤ 20% of Emax reference inducer = “low risk of DDI”; <20–40 < % of Emax reference inducer = “moderate risk of DDI; ≥ 40% of Emax reference inducer = “high risk of DDI”. For CYP1A2 and CYP2B6: if the highest individual value is ≤ 40% of Emax reference inducer = “low risk of DDI "; >40% of Emax reference inducer = “moderate risk of DDI “.

Only CYP induction results obtained at non-cytotoxic concentrations can be reported. To detect possible cytotoxic effect of the compounds under investigation, a microscopic examination is performed. The variation (decrease) of the housekeeping gene is also evaluated (only when mRNA quantification is performed with bDNA technique). The cytotoxicity assessment is performed on the same wells that induction measurement. For calculations, these control conditions are used: 100 %-cell viability, i.e. cells treated with solvent (DMSO), 0 %-cell viability, i.e. 3-fold the background (corresponding to our historical data obtained with 100 μM Menadione). Results are expressed as the percentage of viable cells in treated hepatocytes, relative to control conditions. The TC50 values (concentration corresponding to 50% of housekeeping gene decrease) are determined using the BIOST@T-SPEED software. If a cytotoxicity is observed, a TC50 value will be reported. In absence of cytotoxicity, ≥ higher concentration tested will be reported.

### 7.5 Intrinsic clearance (Clint) in human hepatocytes

The standard conditions clearance study is performed using 5 μM for compound concentration, in the absence or the presence of a specific and potent CYP3A inhibitor: Ketoconazole (3μM). For other CYP suspected to be involved in the metabolism; additional specific and potent inhibitors can be tested, like for example furafylline (1μM) for CYP1A2 inhibition, sulfaphenazole (10μM) for CYP2C9 inhibition, quinidine (3μM) for CYP2D6 inhibition, aminobenzotriazole (1mM) for all CYP isoforms inhibition. Validated cryopreserved as well as fresh human hepatocytes can be used. The source can be “in house” preparations from fresh liver resection obtained from collaboration, or commercially purchased. Human hepatocytes are plated in 48 wells collagen-coated plates and cultivated overnight in appropriate culture medium. Test compounds and inhibitors are prepared as 1000 X stock solution diluted in DMSO and diluted in culture medium (supplemented with 1 % BSA for the test compound solutions). The disappearance kinetics of the test compounds are performed over 24 hours with 8 sampling times: 1, 2, 3, 4, 6, 8 and 24 hours (24h in duplicate) after test compound addition. Incubations are stopped at each sampling time with ACN/Water (50/50 final). Cells are scraped with a cell scraper. Intra- and extra cellular compartments are collected and samples are frozen at −20 °C until analyze. They can be collected pooled (standard experiments) or separately for cellular distribution determination. After thawing, samples are sonicated and proteins are removed by centrifugation. Supernatant fluids are analyzed by HPLC coupled with MS(-MS) detection.

The intrinsic clearance value is calculated according to the following equation:

Clint in vitro = ke. V (written as Clint (Ke 0–24H) in BAR)

where Clint in vitro, is the in vitro intrinsic clearance expressed in mL/h/106 hepatocytes (or cells), ke is the elimination rate constant expressed in h-1 calculated with WinNonlin, via a compartmental analysis, modelling an intravenous bolus injection with no lag time (first order elimination), V is the incubation volume expressed in mL normalized to 10^6^ hepatocytes.

### 7.6 Animals

Male Balb/C mice weighing 25–30 g (Charles River Laboratories) were housed individually in an enriched environment in a pathogen-free facility at a constant temperature of 22 ± 2 °C and humidity (50 ± 10%) on a 12-h light/dark cycle with ad libitum access to food and water. Experiments were performed at Sanofi in full compliance with standards for the care and use of laboratory animals, according to French and European Community legislation. All procedures were approved by the local Animal Ethics Committee and the French Ministry for Research.

### 7.7 Nurr1 agonist and LPS treatment paradigms

The Nurr1 agonist (**cpd 38**) was dissolved in 0.6% methylcellulose and 0.5% Tween-80 in distilled water. 0.6% methylcellulose and 0.5% Tween-80 in distilled water was used as the vehicle. LPS (E Coli; 0111:B04) was dissolved in saline (0.9% sodium chloride). All mice (N = 20) received an intraperitoneal injection of LPS (20 μg/mouse, 10mL/Kg). Thirty minutes before LPS administration, **cpd 38** was administered by oral gavage at dose of 3, 10, or 30 mg/kg (10mL/Kg, N = 5 mice per dose); an additional group of 5 mice were treated with the vehicle (10mL/Kg).

### 7.8 Blood sampling and ELISA quantification of TNFa

Serum samples were collected at 1.5 hours post LPS treatment (and 2 hours post **cpd 38** treatment). Mice were anesthetized with a mixture of ketamine, xylazin 2%, atropine, and saline (4:2.5:1:2.5). Blood samples were collected from mice and allow to clot for 2 hours at room temperature before centrifuging for 20 minutes at 2000×*g*, and sera were removed and stored at −80 °C for the evaluation of TNF-alpha. Serum TNF-alpha levels were analyzed by quantitative sandwich enzyme immunoassay technique (R&D systems) according to manufacturer's instructions. https://www.rndsystems.com/products/mouse-tnf-alpha-quantikine-elisa-kit_mta00b.

R&D Systems: Principle of the assay. This assay employs the quantitative sandwich enzyme immunoassay technique. A monoclonal antibody specific for mouse TNF-α has been pre-coated onto a microplate. Standards, control, and samples are pipetted into the wells and any TNF-α present is bound by the immobilized antibody. After washing away any unbound substances, an enzyme-linked polyclonal antibody specific for mouse TNF-α is added to the wells. Following a wash to remove any unbound antibody-enzyme reagent, a substrate solution is added to the wells. The enzyme reaction yields a blue product that turns yellow when the Stop Solution is added. The intensity of the color measured is in proportion to the amount of TNF-α bound in the initial step. The sample values are then read off the standard curve.

## 8 Safety pharmacology studies

### 8.1 Hemodynamic, electrocardiographic and electrophysiological effects in anesthetized mongrel dogs

#### 8.1.1 Animal preparation

Six fasted adult mongrel dogs (weighing 18–26 kg) were anesthetized with 20 mg/kg sodium thiopental i.v. Animals were artificially ventilated with a gaseous mixture composed of O^2^/air supplemented with around 1.5% end-tidal fraction of isoflurane. Tidal volume and respiratory rate was set at 20 mL/kg and 16 breaths/min. Arterial blood gas was controlled and respiratory adjusted to maintain pH between 7.36 and 7.44, PaO^2^ > 80 mmHg and PaCO^2^ < 40 mmHg. Four limbs electrodes were positioned to obtain a surface ECG. Two bipolar electrodes were positioned in the right atrium via a jugular access, one to record the atrial electrogram and the second one to stimulate the atrium. A bipolar electrode and a quadripolar electrode were inserted via venous and arterial femoral access and positioned in the right ventricular apex and in the His bundle region, respectively. These electrodes were connected to a programmable stimulator and to a computerized physiograph system. In addition, a percutaneous arterial sheath was positioned in the femoral artery in order to record both peripheral blood pressure and left ventricular pressure via a microtip catheter connected to the computerized data acquisition system. A femoral venous catheter was inserted for drug administration.

The auricular diastolic stimulation threshold was evaluated with rectangular current pulse (2 ms duration) increased in steps of 0.1 mA and delivered at fixed rate pacing (S1S1) of 400 or 350 ms in function of animal basal cycle length. If animal cycle length was <350 ms, it was excluded from the study.

#### 8.1.2 Groups and treatment

**Cpd 38** was dissolved in a 75% PEG400 aqueous solution and administered to dogs (n = 3) at 10 mg/kg as a single 5-min intravenous infusion under a volume of 0.5 mL/kg. Control animals (n = 3) received the vehicle only, under the same experimental conditions.

#### 8.1.3 Parameters monitored

After a 30-min stabilization period, the auricular stimulation threshold was verified and the following parameters were evaluated before drug administration (baseline) and then:

- at 5, 10 and 50 minutes after the beginning of the drug infusion for ECG intervals (RR, PQ, QRS, QT intervals (ms); the QTc interval was calculated using the Bazett's and Fridericia formulas), - at 5, 10, 20, 30, 40, 50, 60 minutes after the beginning of the drug infusion for hemodynamic parameters (Heart rate (bpm); Diastolic, mean, systolic arterial pressure (mmHg); Left ventricular pressure and ventricular contractility).
- at 10 and 50 minutes after the beginning of the drug infusion for electrophysiological parameters (Wenckebach cycle length (ms), Atrial effective refractory period (ms), Ventricular effective refractory period (ms)).

#### 8.1.4 Plasma and tissue drug concentration determination

Blood samples were drawn from a femoral artery at 10 and 60 minutes after the beginning of the drug perfusion and after centrifugation plasma samples were stored frozen at −20 °C until analysis.

At the end of the experiment (around 60 minutes following the beginning of the drug perfusion), the heart was removed and a few grams of tissue were collected from right and left ventricles stored frozen at - 20 °C until analysis.

Analysis of samples was performed using an exploratory LC-MS-MS assay method. Plasma (LOQ of 2 ng/mL) and tissue (LOQ of 3 ng/g) concentrations of **Cpd 38** were determined.

Results: No major hemodynamic, ECG and electrophysiological effects were observed.

## 8.2 Hemodynamic and electrocardiographic effects in anesthetized male hartley Guinea pigs

### 8.2.1 Animal preparation

Male Hartley guinea pigs (weighing 400–500 g) were anesthetized with 35 mg/kg sodium pentobarbital i.p. and let under spontaneous breathing conditions. The body temperature was maintained at 37.5 ± 0.5 °C throughout the experiment. For implantation of vascular catheters, cutaneous incision, subcutaneous and muscular dissections were performed after application of local anesthetic (lidocain, 20 mg/kg). A carotid artery and a jugular vein were catheterized to allow recording of arterial blood pressure and intravenous administration of the drug, respectively. The arterial micromanometer catheter was connected to a pressure transducer. For the electrocardiogram recording, five electrodes were inserted in the subcutaneous layer of fore- and hind-limb. The pressure transducer and ECG electrodes were linked to Gould pre-amplifiers. The amplifiers were connected to a Notocord-Hem computerized data acquisition system and recorded at a sampling rate of 1000 Hz. Either lead I or lead II were recorded, depending on which the better separation of T wave from P wave of the next complex was achieved.

### 8.2.2 Groups and treatment

**Cpd 38** was dissolved in a 75% PEG400 aqueous solution and administered to guinea pigs (n = 6) at 10 mg/kg as a single 5-min intravenous infusion under a volume of 0.5 mL/kg. Control animals (n = 10) received the vehicle only, under the same experimental conditions.

### 8.2.3 Parameters monitored

The following parameters were evaluated before drug administration (baseline corresponding to the average of 3 values taken at −2, −1 and 0 minute before starting the infusion), then every each minute post-beginning of the infusion for 5 minutes, and then every 5 minutes until 30 minutes post-beginning of infusion.

- ECG intervals (RR, PR, QRS, QT intervals (ms); the QTc interval was calculated using the Bazett's formula).
- hemodynamic parameters (Heart rate (bpm); mean arterial pressure (mmHg)).

### 8.2.4 Plasma and tissue drug concentration determination

Blood samples were drawn from a carotid artery at 10 and 30 minutes after the beginning of the drug perfusion and after centrifugation plasma samples were stored frozen until analysis.

At the end of the experiment (around 30 minutes following the beginning of the drug perfusion), the heart was removed and a few grams of tissue were collected from right and left ventricles stored frozen at - 20 °C until analysis.

Analysis of samples was performed using an exploratory LC-MS-MS assay method. Plasma (LOQ of 2 ng/mL) and tissue (LOQ of 10 ng/g) concentrations of **Cpd 38** (expressed as active ingredient).were determined.

Results: No major hemodynamic and ECG effects were observed.
