# Supplementary material for: Data on synthesis, ADME and pharmacological properties and early safety pharmacology evaluation of a series of novel NURR1/NOT agonist potentially useful for the treatment of Parkinson's disease
Source: Data Brief. 2019 May 27;27:104057. doi: 10.1016/j.dib.2019.104057 (PMC6820105; doi:10.1016/j.dib.2019.104057)
Supplement: Multimedia component 1 [file mmc1.doc]

Dominique LESUISSE, PhD

Head of CNS Barriers

Rare and Neurologic Diseases Research TA

SANOFI

1 av Pierre Brossolette

Chilly Mazarin, F-91935

TEL.: +33 (0) 1.60.49.68.57 - CELL.: +33 (0) 6.88.06.92.70

To whoever it may concern :

February 14th, 2019.

Hereby I, Dominique Lesuisse certify that no potential competing interest or undisclosed funcing source exist for this manuscript.

I thank you very much in advance and remain

Sincerely yours,

Dominique Lesuisse
